# Effects of insemination and blood-feeding on locomotor activity of *Aedes albopictus* and *Aedes aegypti* (Diptera: Culicidae) females under laboratory conditions

**DOI:** 10.1186/1756-3305-7-304

**Published:** 2014-07-02

**Authors:** Tamara Nunes Lima-Camara, José Bento Pereira Lima, Rafaela Vieira Bruno, Alexandre Afranio Peixoto

**Affiliations:** 1 Programa de Computação Científica – PROCC. Fundação Oswaldo Cruz/FIOCRUZ, Av. Brazil, 4365, Manguinhos, Rio de Janeiro CEP 21040-360, Brazil; 2 Laboratório de Biologia Molecular de Insetos, Instituto Oswaldo Cruz, IOC-FIOCRUZ, Av. Brazil, 4365, Manguinhos, Rio de Janeiro CEP 21040-360, Brazil; 3 Departamento de Epidemiologia da Faculdade de Saúde Pública da USP, Arnaldo, 715, Cerqueira César, São Paulo CEP - 01246-904, Brazil; 4 Laboratório de Fisiologia e Controle de Artrópodes Vetores, Instituto Oswaldo Cruz, IOC-FIOCRUZ, Av. Brazil, 4365, Manguinhos, Rio de Janeiro CEP 21040-360, Brazil; 5 IBEX – Instituto de Biologia do Exército, Rua Francisco Manuel, 102, Benfica, Rio de Janeiro CEP 20911-270, Brazil; 6 Instituto Nacional de Ciência e Tecnologia em Entomologia Molecular (INCT-EM)/CNPq, Rio de Janeiro, Brazil

**Keywords:** *Aedes albopictus*, *Aedes aegypti*, Physiology, Locomotor activity, Laboratory

## Abstract

**Background:**

Dengue is an arbovirus disease transmitted by two *Aedes* mosquitoes: *Ae. aegypti* and *Ae. albopictus*. Virgin females of these two species generally show a bimodal and diurnal pattern of activity, with early morning and late afternoon peaks. Although some studies on the flight activity of virgin, inseminated and blood-fed *Ae. aegypti* females have been carried out under laboratory conditions, little is known about the effects of such physiological states on the locomotor activity of *Ae. albopictus* and *Ae. aegypti* females. The aim of this study was to analyze, under laboratory conditions, the effects of insemination and blood-feeding on the locomotor activity of *Ae. albopictus* and *Ae. aegypti* females under LD 12:12, at 25°C.

**Methods:**

Both *Ae. albopictus* and *Ae. aegypti* females were obtained from established laboratory colonies. Control groups were represented by virgin/unfed *Ae. albopictus* and *Ae. aegypti* females. Experiments were conducted under laboratory conditions, using an activity monitor that registers individual activity every thirty minutes.

**Results:**

Virgin/unfed *Ae. albopictus* and *Ae. aegypti* females showed a diurnal and bimodal pattern of locomotor activity, with peaks at early morning and late afternoon. Insemination and blood-feeding significantly decreased the locomotor activity of *Ae. aegypti* females, but inseminated/blood-fed *Ae. aegypti* and *Ae. albopictus* females showed a similar significant decrease on the locomotor activity compared to virgin/unfed females.

**Conclusions:**

This study is the first demonstration of the effects of insemination and blood-feeding on the locomotor activity of *Ae. albopictus* and *Ae. aegypti* females under artificial conditions. Data suggest that *Ae. albopictus* and *Ae. aegypti* females respond in different ways to physiological status changes and such divergence between these two dengue vectors, associated with several ecological differences, could be related to the greater dengue vectorial capacity of *Ae. aegypti* in Americas in comparison to *Ae. albopictus*.

## Background

Dengue is an arbovirus disease predicted to be ubiquitous throughout the tropics and it is strongly influenced by temperature, rainfall and high urbanization. Recently, 96 million apparent infections and 293 million unapparent infections of dengue cases were estimated in the world per year [1]. *Aedes (Stegomyia) aegypti* (Linnaeus 1762) is considered the main dengue vector in the world and has a wide distribution in tropical countries. *Aedes (Stegomyia) albopictus* (Skuse 1994) originated in Asia, where it can play an important role as a dengue vector, and also has a wide distribution in tropical as well as temperate countries [2]. In Brazil, as well as in a few countries, *Ae. albopictus* is considered a potential vector of dengue and other arboviruses, such as yellow fever and Chikungunyia [3-5].

Daily rhythms in behaviours, such as flight, mating, blood-feeding and oviposition are observed in mosquitoes, which restrict their activities to specific hours of the day. This is due to an endogenous circadian clock that can be synchronized to external cues, such as light, temperature and food, among others [6]. In insect vectors, the relationship between the circadian clock and the different behaviours are particularly important to their vectorial capacity. Besides, the different physiological states of the individuals can modulate the way they respond to several stimuli. For instance, in the sandfly *Lutzomyia longipalpis*, the main vector of American Visceral Leishmaniasis, blood-fed females present a significant decrease of their activity pattern in comparison to the unfed ones [7]. In *Anopheles gambiae*, the main malaria vector in Africa, circadian rhythmicity drives inseminated females to engage in host-seeking flight characterised by a broad peak of activity throughout the night [8], in contrast to the bimodal activity of virgin females seeking mating swarms [9]. When mosquitoes are unfed, the host-seeking behaviour can be modified by host-associated stimuli [10].

According to their locomotor activity pattern, *Ae. aegypti* and *Ae. albopictus* are considered diurnal vectors, with more activity during the early morning and the late afternoon under natural conditions [2,11,12]. In general, virgin *Ae. aegypti* and *Ae. albopictus* males and females copulate two or three days after emergence and inseminated females seek a vertebrate host to blood-feed and develop eggs [13-15]. Insemination and blood-feeding are two factors that might affect the behaviour of *Ae. aegypti* and *Ae. albopictus* females. In fact, the accessory glands of *Aedes* male mosquitoes produce substances that are transferred to females during mating that can influence their circadian rhythmicity and also alter some aspects of female physiology and behaviour, such as mating, preoviposition and host-seeking behaviours [16,17]. Under laboratory conditions, the flight activity of virgin, inseminated and blood-fed *Ae. aegypti* females has been evaluated [18]. The author reported significant differences on the total flight activity of the specimens and showed that it is important to analyze the pattern of activity of virgin females as well as inseminated and blood-fed ones [18].

Although some studies have been done on the flight activity of *Ae. aegypti* females in different physiological states [19], little is known about the locomotor activity of inseminated and/or blood-fed *Ae. albopictus* and *Ae. aegypti* females in artificial conditions. Thus, the aim of this study was to evaluate, under laboratory conditions, the effect of insemination and blood-feeding on the locomotor activity of *Ae. albopictus* and *Ae. aegypti* females.

## Methods

### Mosquito rearing

*Ae. aegypti* Rockefeller strain adult mosquitoes were obtained from colonies of LAFICAVE since 1986 (Laboratório de Fisiologia e Controle de Artrópodes Vetores, Fiocruz, Rio de Janeiro, Brazil), whereas *Ae. albopictus* adults were obtained from colonies established in LATHEMA (Laboratório de Transmissores de Hematozoários, Fiocruz, Rio de Janeiro, Brazil). Immature forms were raised in trays with tap water and fish food (Tetramin®) [2], under LD 12:12, at 25°C. Pupae of both species were isolated individually to ensure virgin males and females after emergence. For experiments with inseminated females, approximately 50 three-days old females were placed into a cage with approximately 100 males of the same age, for 24 hours.

For experiments with blood-fed females, 4-5-day old females were fed on anesthetized Guinea pig (ketamine:xylazine 80–120:10–16 mg/kg) for 2 hours, according to institutional procedures, oriented by the national guideline ‘the Brazilian legal framework on the scientific use of animals’ [20]. Locomotor activity analysis started at 24 hours after insemination and 2 hours after blood feeding.

Cotton soaked in a 15% sucrose solution was continuously available for all mosquitoes as a sugar source, except for experiments with blood-fed females, when sucrose solution was taken out 24 hours before blood-feeding. All cages with *Aedes* adults were placed inside the Precision Scientific Incubator Mod. 818, under same experimental photoperiod, humidity and temperature conditions as the raising phase. Spermathecae of all inseminated females were dissected at the end of locomotor activity experiments to ensure insemination.

### Analysis of locomotor activity

Locomotor activity pattern of virgin/unfed (control group), inseminated/unfed, virgin/blood-fed and inseminated/blood-fed *Ae. aegypti* females was evaluated with a Locomotor Activity Monitor (TriKinetics), first developed for *Drosophila*. Locomotor activity is one of the 24 hour scale rhythms that can be monitored over many days. Adult females are placed into small glass tubes and those are placed into special trays equipped with infrared light and detectors. By introducing the infrared beam into the path of the mosquito, a computer counts every time the beam is broken, giving a measure of the pattern of activity and rest of the mosquito [21].

Four up to five - day -old adults were individually placed in glass tubes (10 mm × 7 cm) with a cotton plug soaked in 15% sucrose solution and these tubes placed in a Locomotor Activity Monitor inside a Precision Scientific Incubator Mod. 818 under a constant temperature of 25°C and a photoperiod of 12 hours of light and 12 hours of dark (LD 12:12), as previously described [22-24]. All locomotor activity experiments started with 32 individuals.

For every mosquito, 48 data points representing the total locomotor activity of 30 minute intervals were obtained for every day of monitoring. All females that survived the first 3 days of experiment were analyzed. We calculated the Williams mean (Wm) as an estimate of the central tendency of activity during each time interval. The Williams mean is a modified geometric mean that accommodates frequent zero values [25].

### Statistical analysis

For statistical analyses, we first tested the normality of all data using the Kolmogorov-Smirnov test. Since the normality assumptions were satisfied, we used the parametric independent samples T-test.

The daily locomotor activity was summarized by seven indices: (1) the *total activity mean*; (2) the *diurnal activity mean*; (3) the *diurnal activity without lights-on mean*, i.e., the mean activity during the photophase except for the first 30 minutes, which corresponds to the morning peak, (4) the *lights-on mean*, which corresponds to the first 30 minutes just after the lights-on, (5) the *nocturnal activity mean*, (6) the *nocturnal activity without lights-off mean*, i.e., the mean activity during the scotophase except for the first 30 minutes, which corresponds to the evening peak and (7) the *lights-off mean*, which corresponds to the first 30 minutes just after the lights-off.

## Results

**Figure 1:**
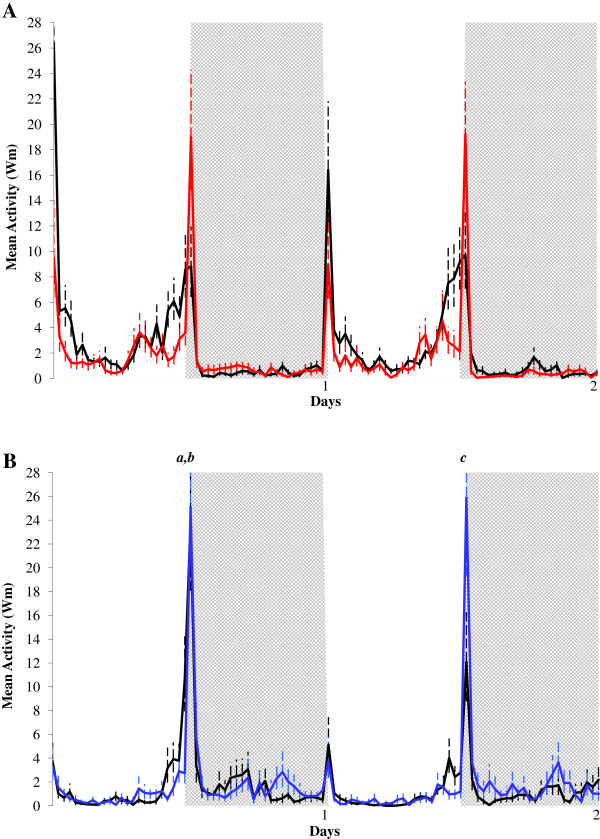
**Effects of insemination on the locomotor activity of *Aedes aegypti *(A) and *Aedes albopictus *(B).** Locomotor activity of virgin/unfed (black line; n = 32 for *Ae. aegypti* and n = 24 for *Ae. albopictus*) and of inseminated/unfed (red line) *Ae. aegypti* females (**A**; n = 32) and (blue line) *Ae. albopictus* females (**B**; n = 24) exposed to two days of LD 12:12, at 25°C. First and second days represent, respectively, 48 and 72 hours after insemination. White column represents the photophase and grey column the scothophase. Some reading time points exceeding the scale are indicated with letters, and their values are as follows: *a* (virgin/unfed) = 23.97 (+7.69); *b* (inseminated/unfed) = 25.15 (+6.61); *c* = 25.90 (+8.68).

Figure 1 shows the locomotor activity of inseminated/unfed *Ae. aegypti* (A; red line) and *Ae. albopictus* (B; blue line) females compared to the respective control groups (virgin/unfed females – black lines) under two days of LD 12:12, at 25°C. First day in the graph represents the next day after blood-feeding (24 h) and two days after insemination (48 hours).

In general, *Ae. aegypti* (Figure 1A) and *Ae. albopictus* (Figure 1B) control females (virgin/unfed) showed a diurnal and bimodal pattern of activity, with peaks at lights-on and lights-off, and more activity at the end of photophase, in a characteristic nocturnal anticipation. Inseminated/unfed *Ae. aegypti* (Figure 1A; red line ) and *Ae. albopictus* females (Figure 1B; blue line) also showed a diurnal and bimodal pattern of activity, with smaller peaks at lights-on and higher peak at lights-off compared to the control group (Figures 1A and B; black lines). Inseminated/unfed *Ae. aegypti* females showed significant decreases in the diurnal mean (t = 2.567; df = 62; p = 0.013), diurnal mean without lights-on (t = 2.417; df = 62; p = 0.019), lights-on mean (t = 2.195; df = 50.549; p = 0.033) and lights-off mean (t = −2.305; df = 55.327; p = 0.025) compared to control group. Nevertheless, no significant decrease was observed in any of the parameters evaluated for inseminated/unfed *Ae. albopictus* females compared to virgin/unfed *Ae. albopictus* females.

**Figure 2:**
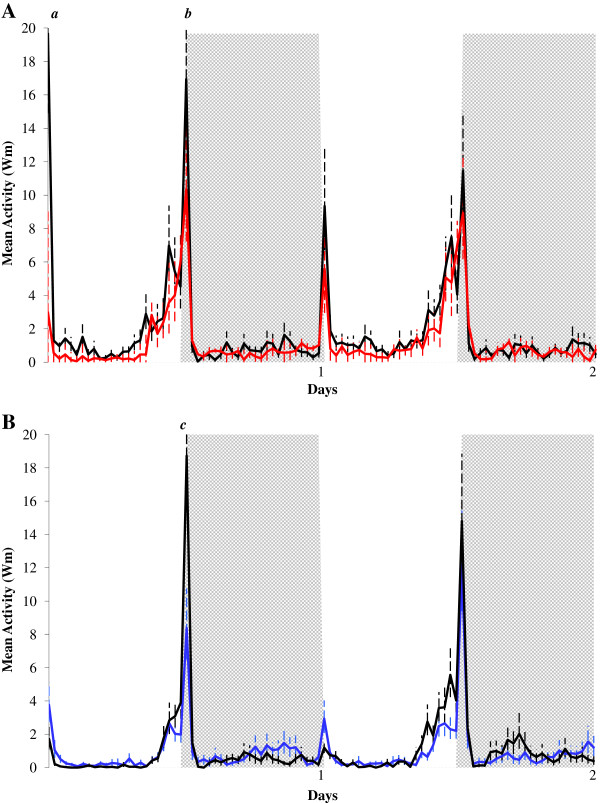
**Effects of blood-feeding on the locomotor activity of *Aedes aegypti *(A) and *Aedes albopictus *(B).** Locomotor activity of virgin/unfed (black line; n = 32 for *Ae. aegypti* and n = 28 for *Ae. albopictus*) and of virgin/blood-fed (red line) *Ae. aegypti* females (**A**; n = 29) and (blue line) *Ae. albopictus* females (**B**; n = 28) exposed to two days of LD 12:12, at 25°C. First and second days represent, respectively, 24 and 48 hours after blood-feeding. White column represents the photophase and grey column the scothophase. Some reading time points exceeding the scale are indicated with letters, and their values are as follows: *a* = 19.66 (+6.15); *b* = 16.93 (+3.77); *c* = 18.72 (+3.86).

Figure 2 shows the locomotor activity of virgin/blood-fed *Ae. aegypti* (A; red line) and *Ae. albopictus* (B; blue line) females compared to the respective control groups (virgin/unfed females – black lines) under two days of LD 12:12, at 25°C. First day in the graph represents the next day after blood-feeding (24 h) and two days after insemination (48 hours). *Ae. aegypti* (Figure 2A) and *Ae. albopictus* (Figure 2B) control females (virgin/unfed; black lines) showed a diurnal and bimodal pattern of activity, with peaks at lights-on and lights-off, and more activity at the end of photophase (Figures 2A and B). Virgin/blood-fed *Ae. aegypti* (Figure 2A; red line) and *Ae. albopictus* females (Figure 2B; blue line) also showed a diurnal and bimodal pattern of activity, but lights-on and lights-off peaks of *Ae. aegypti* control females were higher than virgin/blood-fed *Ae. aegypti* females (Figure 2A), whereas virgin/blood-fed *Ae. albopictus* females showed a smaller lights-on peak and a higher lights-off peak compared to virgin/unfed *Ae. albopictus* females (Figure 2B).

Compared to virgin/unfed *Ae. aegypti* females, virgin/blood-fed *Ae. aegypti* females showed a significant decrease in the mean activity of two days (t = 2.553; df = 59; p = 0.013), diurnal mean (t = 4.239; df = 59; p < 0.001), diurnal mean without lights-on (t = 3.931; df = 59; p < 0.001) and lights-on mean (t = 3.761; df = 59; p < 0.001). On the other hand, except for the lights-on mean (t = 2.098; df = 54; p = 0.041), similar to inseminated/unfed *Ae. albopictus* females, virgin/blood-fed *Ae. albopictus* females showed no significant decrease in any of the parameters evaluated when compared to virgin/unfed females.

**Figure 3:**
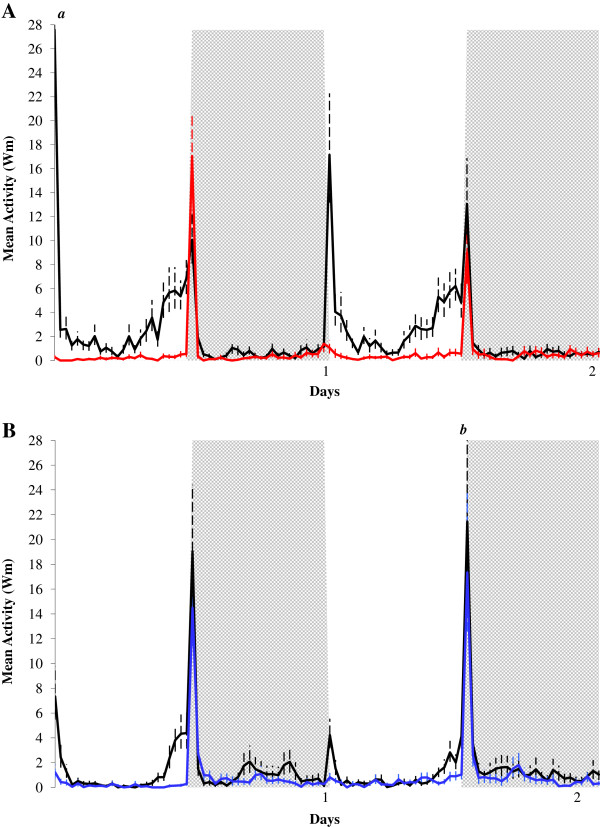
**Effects of insemination and blood-feeding on the locomotor activity of *Aedes aegypti *(A) and *Aedes albopictus *(B).** Locomotor activity of virgin/unfed (black line; n = 31 for *Ae. aegypti* and n = 28 for *Ae. albopictus*) and of inseminated/blood-fed (red line) *Ae. aegypti* females (**A**; n = 25) and (blue line) *Ae. albopictus* females (**B**; n = 26) exposed to two days of LD 12:12, at 25°C. First and second days represent, respectively, 48 and 72 hours after insemination and 24 and 48 hours after blood-feeding. White column represents the photophase and grey column the scothophase. Some reading time points exceeding the scale are indicated with letters, and their values are as follows: *a* = 27.57 (+6.81); *b* = 21.44 (+6.79).

As verified in control females of Figures 1 and 2, the locomotor activity of control *Ae. aegypti* and *Ae. albopictus* females (virgin/unfed) (Figure 3) was also diurnal, with more activity at the end of photophase and bimodal, with peaks at lights-on and lights-off, generally higher than inseminated/blood-fed *Ae. aegypti* (Figure 3A; red line) and *Ae. albopictus* (Figure 3B; blue line) females. Inseminated/blood-fed *Ae. aegypti* (Figure 3A; red line) and *Ae. albopictus* (Figure 3B; blue line) females showed very low activity in the two tested days, showing almost no lights-on peak and activity in the end of photophase. The lights-off peaks were higher in both inseminated/blood-fed *Ae. aegypti* and *Ae. albopictus* females compared to control females (Figure 3). Significant decreases in the total activity mean (t = 8.508; df = 48.196; p < 0.001), diurnal mean (t = 9.705; df = 48.790; p < 0.001), diurnal mean without lights-on (t = 8.930; df = 48.498; p < 0.001) and lights-on mean (t = 11.479; df = 54; p < 0.001) were verified for inseminated/blood-fed *Ae. aegypti* females compared to the virgin/unfed *Ae. aegypti* females. Similar significant decreases were reported for the total activity mean (t = 3.312; df = 43.298; p = 0.002), diurnal mean (t = 4.123; df = 52; p < 0.001), diurnal mean without lights-on (t = 3.657; df = 52; p = 0.001) and lights-on mean (t = 4.652; df = 52; p < 0.001) were verified for inseminated/blood-fed *Ae. albopictus* females compared to the virgin/unfed *Ae. albopictus* females.

## Discussion

Circadian rhythms have been extensively studied in insects due to their importance in regulating many behaviours, especially in vector species. Unlike the model *Drosophila melanogaster*, which comprises behavioural and genetic evidences of the regulation of these rhythms, only recently studies on insect vectors are shedding light on this issue.

In *Anopheles gambiae*, an inhibition in blood feeding was observed in females with reduced expression of clock genes via RNAi [26], corroborating the idea that clock genes regulate blood feeding. Thus, further transcriptome studies showed classes of genes regulated by the circadian clock, including detoxification, redox and metabolism genes [27]. In the same way, *Lutzomyia longipalpis* also present a robust decrease in the amplitude of the locomotor activity in blood fed females [7].

Bimodal and diurnal rhythms have already been reported for *Ae. aegypti* and *Ae. albopictus* females when testing flight, sugar-feeding and host-seeking activities under laboratory conditions [18,28,29]. In addition, under laboratory conditions, it has been reported that parasite-vector interactions, such as dengue virus- *Ae. aegypti* infection, as well as genetic mutations which denote insecticide resistance by the vector do not change the pattern of activity of *Ae. aegypti*, but increase the locomotor activity of females [24,30]. Although it has been reported that physiological states, such as insemination and blood-feeding, affect the flight activity of *Ae. aegypti* females [18], it has not been evaluated up to now how drastic changes in these physiological states could affect the locomotor activity pattern of *Ae. aegypti* and *Ae. albopictus* females.

In general, *Ae. aegypti* females show great alterations in their pattern of activity when they are inseminated, because male accessory gland (MAG) produces some peptides and other elements that are transferred to females during copulation and modulate their host-seeking behaviour [16,17]. It is also known that *Aedes albopictus* MAG is able to decrease the diurnal locomotor activity of *Ae. aegypti* females in a similar way to *Ae. aegypti* MAG, making them refractory to mating with conspecific males [31]. This refractoriness may lead to a reduction in the vectorial capacity of both species. Such modifications have also been described in other mosquito species, including *An. gambiae*[8,9] and *An. stephensi*[32].

In this study, we observed that inseminated/unfed *Ae. aegypti* females showed a significant decrease in the locomotor activity compared to virgin/unfed females, whereas inseminated/unfed *Ae. albopictus* females did not show significant decrease in the locomotor activity compared to the control group. This could be explained by the ecological needs of each species. It is well known that *Ae. aegypti* has a close association with human houses, where they mate and blood-feed [18,19]. Thus, after mating, *Ae. aegypti* females do not need to be active for a long period to find a vertebrate and blood-feed [33]. On the other hand, *Ae. albopictus* is a more sylvatic species [2], needing to be active for longer periods to mate and, then, seek a vertebrate to blood-feed. A similar decrease in flight activity of inseminated/unfed *Ae. aegypti* females has been reported under laboratory conditions [18], but no previous data have shown the effect of insemination on the activity of *Aedes albopictus* females.

A fully engorged female is much heavier than an unfed one and it has been reported that abdominal distention by blood feeding or developing ovaries can inhibit the activity of host-seeking of *Ae. aegypti* females [34,35]. Thus, until eggs are fully developed and ready to be laid, we expect a decrease in locomotor activity in both engorged *Aedes* females, but it was only observed in *Ae. aegypti* females. Once more, the explanation for this difference could rely on the ecology of each species, as mentioned above. In addition, under laboratory conditions, it has been demonstrated that insemination and blood-feeding can affect the circadian expression of specific genes involved in circadian clocks and, consequently, in circadian rhythms of *Ae. aegypti* and other hematophagous vectors [7,35]. In the opposite way, when clock gene expression is reduced, the blood feeding is inhibited, indicating that not only the clock genes expression *per se* but also the clock controlled genes (ccg´s) are fundamental for the blood feeding behaviour [26]. The circadian expression of such specific genes that control circadian rhythms in inseminated and blood-fed *Ae. albopictus* females deserves further investigations.

Inseminated/blood-fed *Ae. aegypti* and *Ae. albopictus* females showed similar decreases in locomotion compared to virgin/unfed *Ae. aegypti* and *Ae. albopictus.* Under natural conditions, 2–3 days after being inseminated, *Ae. aegypti* and *Ae. albopictus* females seek a vertebrate host to blood-feed [14,15]. Blood digestion triggers ovarian development and females are considered gravid and ready to oviposit three days later [36]. Thus, we can expect low activities of inseminated/blood-fed *Ae. aegypti* and *Ae. albopictus* females in the first two days since eggs are already being developed to be oviposited about three days later.

Except for the lights-off mean of inseminated/unfed *Ae. aegypti* females, neither insemination nor blood-feeding significantly affected the nocturnal mean, the nocturnal mean without lights-off and the lights-off mean of both species compared to the virgin/unfed females. This data can be explained by the frequent diurnal behaviour shown by *Ae. aegypti* and *Ae. albopictus* under natural conditions, flying, mating and blood-feeding preferentially during the early morning and late afternoon hours [37].

## Conclusions

This study is the first demonstration of the effects of insemination and blood-feeding on the locomotor activity of *Ae. albopictus* and *Ae. aegypti* females under artificial conditions. Since we have compared the behaviour of *Ae. albopictus* and *Ae. aegypti* females from laboratory colonies, it would be helpful if insemination and blood-feeding effects could also be studied with F1 or F2 generations of these mosquitoes. Nevertheless, this study observed several differences between the effects of insemination and blood-feeding on these two dengue vector females that, associated with some ecological differences between both species, could be related to the better dengue vectorial capacity of *Ae. aegypti* in Americas in comparison to *Ae. albopictus*. Thus, further studies of insemination and blood-feeding effects on *Ae. albopictus* and *Ae. aegypti* females are necessary to fully understand the biology and behaviour of these two important dengue vectors.

## Competing interests

The authors declare that they have no competing interests.

## Authors’ contributions

TNLC and AAP designed the study. JBPL raised the mosquito larvae and carried out the blood-feeding of females. TNLC, AAP and RVB analyzed and interpreted all locomotor activity data. AAP supervised all laboratory experiments, analysis of the data and interpretation of results. TNLC and RVB were involved in drafting the manuscript. All authors approved the final version of the manuscript.
